# Granulocytes mediates the Fas-L-associated apoptosis during lung metastasis of melanoma that determines the metastatic behaviour

**DOI:** 10.1038/sj.bjc.6600461

**Published:** 2002-08-01

**Authors:** Y-L Chen, J-Y Wang, S-H Chen, B-C Yang

**Affiliations:** Department of Microbiology and Immunology, College of Medicine, National Cheng Kung University, 138 Sheng Li Road, Tainan 740, Taiwan, Republic of China; Department of Pediatics, College of Medicine, National Cheng Kung University, 138 Sheng Li Road, Tainan 740, Taiwan, Republic of China

**Keywords:** lung metastasis, Fas ligand, apoptosis, granulocyte

## Abstract

The survival of tumour cells in a new tissue environment is crucial for tumour metastasis. Factors contributing to the death of tumour cells during metastasis are not completely understood. In murine melanoma model, activation of Fas (CD95, APO-1) signal in tumour cells reduces their lung metastasis potential, which may be associated with an induction of apoptosis in tumours. To elucidate the cellular mechanism, we used a Fas-ligand (Fas-L) specific ribozyme (Fas-L^ribozyme^) to suppress the expression of Fas-L but not Fas or TNF-α in B16F10 melanoma cells. The Fas-L^ribozyme^-carrying cells grew slightly faster *in vitro* with better viability than controls. Suppression of Fas-L in B16F10 melanoma cells by Fas-L^ribozyme^ enhanced lung metastasis of the cells in C57BL/6 mice, and that was correlated with reductions in both apoptotic tumour cells and granulocytic infiltration. Mice depleted of granulocytes, but not CD4^+^ and CD8^+^ cells, showed a greatly elevated susceptibility to lung metastasis. Moreover, apoptosis in tumour cells was significantly reduced in granulocyte-depleted mice during the course of tumour formation. Taken together, our findings indicate that Fas-L-associated apoptosis in tumour cells determines the metastasis behaviour of melanoma in the lung and this apoptosis is primarily mediated by the cytotoxicity of recruited granulocytes.

*British Journal of Cancer* (2002) **87**, 359–365. doi:10.1038/sj.bjc.1200461 www.bjcancer.com

© 2002 Cancer Research UK

## 

Apoptosis which occurs in tumour cells during or just after extravasation from blood vessel into a new tissue environment is a crucial step in metastasis (Walsh and Sata, 1999). Apoptotic factors of tumours contributing to metastatic tumour behaviour are not completely understood. Among the candidate apoptotic signals, the Fas (also named as CD95, APO-1)/Fas-ligand (Fas-L) system plays important roles in organ homeostasis and immune surveillance against tumours (Walker *et al*, 1998). Fas-L is a member of the tumour necrosis factor receptor superfamily and triggers a death signal into Fas-bearing cells after engagement with Fas molecule (Itoh *et al*, 1991; Takahashi *et al*, 1994). Functional expression of Fas-L in melanoma has been demonstrated *in vitro* (Hahne *et al*, 1996). During the progression of human melanoma, tumour Fas-L increases gradually (Terheyden *et al*, 1999; Soubrane *et al*, 2000). However, the finding that the Fas/Fas-L interaction suppressed lung metastasis of melanoma in murine models argued against a major contribution of tumour Fas-L in escape from immune surveillance (Owen-Schaub *et al*, 1998; Rivoltini *et al*, 1998; Sprecher *et al*, 1999) and suggested a negative role of Fas-associated apoptosis in metastasis. In addition, ectopic expression of Fas-L in transgenic animals or tumours revealed that Fas-L enhanced neutrophil recruitment and mediated destruction of certain Fas-L-positive cells (Arai *et al*, 1997; Chervonsky *et al*, 1997; Seino *et al*, 1997; O'Flaherty *et al*, 1998). At moment, it is not known whether the Fas-L-associated suppression in metastasis is mediated by triggering the suicidal apoptotic Fas signal in tumour cells or by an indirect action through the recruited inflammatory cells.

In this study, we investigated the contribution of immune cells to Fas-associated apoptosis in tumours. We used a hammerhead Fas-L-specific ribozyme (Fas-L^ribozyme^) to suppress the Fas-L gene (Yang *et al*, 1999; Chio *et al*, 2001). The Fas-L^ribozyme^ effectively inhibited the expression of Fas-L in melanoma cells at both mRNA and protein levels. Stable transfectants carrying Fas-L^ribozyme^ were established to evaluate the contribution of tumour Fas-L to cell growth, apoptosis, and lung metastasis. The involvement of immune cells in the processes of lung metastasis was explored by cell depletions using antibodies for CD4-, CD8-cells or granulocytes. Our results clearly demonstrate that granulocytes play a crucial role in the Fas-L-associated apoptosis during lung metastasis.

## MATERIALS AND METHODS

### Cell culture

The melanoma cell line B16F10, kindly provided by Dr Shiau Al, NCKU, is derived from a spontaneous melanoma in C57BL/6 mouse and has lung metastasis ability (Mackensen *et al*, 1993). Tumour cells were cultured in DMEM medium (Life Technologies, Grand Island, NY, USA) supplemented with 10% foetal calf serum (FCS) and 2 mM L-glutamine at 5% CO_2_/37°C in a humidified atmosphere. Cell growth rate was determined. In brief, 2.5×10^4^ of cells in a 60 mm-dish were at first starved in 0.1% FCS/DMEM for 24 h, and then re-grew in a regular 10% FCS/DMEM. Cells were harvested at intervals to determine the number of viable cells by Trypan blue exclusion method.

### DNA transfection and selection of stable cells

The sequences of the oligonucleotides used to construct Fas-L^ribozyme^ were as follow: sense sequence: 5′-ATGAATTCCCGGAAGTACTGATGAGTCGTGATACGACGAAACTTTGGATCCCGA-3′; antisense sequence: 5′-TCGGGATCCAAAGTTTCGCGTATCACGACTCATCAGTACTTCCGGGAATTCAT-3′. The ribozyme was directly linked to upstream of the EGFP gene in pEGFP-N1 plasmid (CLONTEC, Palo Alto, CA, USA) to form a fusion transcript (Chio *et al*, 2001). Plasmid DNA was delivered into cells using the lipofection method with a ratio of 5 μg DNA per 30 μl lipofectamine (Qiagen, Hilden, Germany). Cells transfected with pEGFP-N1 plasmid served as the vector control. After DNA transfection, cells were grown in regular 10% FCS/DMEM for 48 h and then selected with geneticin (G418 sulphate, Gibco, Darmstadt, Germany) at an effective concentration of 1.5 mg ml^−1^. Bulk culture or stable clones were established for at least 3 months before they were subjected to further study.

### Semi-quantitative reversed transcription-polymerase chain reaction (RT–PCR)

Total RNA was purified using the RNeasy Kit according to the manufacturer's instruction (Qiagen) and converted to cDNA by StrataScrip™-H-reverse transcriptase with oligo-dT primer in the presence of RNasin (Stratagen, CA, USA). RT–PCR for Fas-L, Fas, TNF-α and β-actin were performed as described previously (Hahne *et al*, 1996; Yang *et al*, 1999). The cDNA generated was subjected to 25–35 cycles of PCR amplification on a DNA Thermal Cycler (Hybaid Omnigene, Middlesex, UK). β-Actin served as a quantitative control for PCR. PCR products were fractionated by agarose electrophoresis, stained with ethidium bromide, and visualised under UV light.

### Western blot

Cells were extracted with a buffer containing 20 mM Tris, 150 mM NaCl, 1 mM EDTA, 1% Nonidet P-40, 1 mM PMSF and 0.1 U ml^−1^ leupeptin. Proteins were separated in SDS-polyacrylamide gel and electroblotted onto polyvinyl difluoride membrane (MSI, Westboro, MO, USA). The proteins bounded on the membrane were probed with the mouse antibody (Ab)-recognising Fas-L (clone33; Transduction Laboratories, Lexington, KY, USA) followed by a sheep anti-mouse IgG conjugated with horseradish peroxidase (Dako Corp., Carpinteria, CA, USA). The Fas-L band was made visible by fluorography with enhanced chemiluminescence detection kit (Amersham/Pharmacia Biotech., UK). Duplicate blot was probed with α-tubulin-specific Ab (clone DM1A; NeoMarker, Fremont, CA, USA) and served as protein-loading control.

### Tumour formation and immunohistochemical staining

Eight-week-old C57BL/6 mice (H2^b^) were purchased from the National Laboratory Animal Breeding and Research Center, Taiwan, R.O.C. and maintained under specific pathogen-free condition. All animal experiments have been carried out with approval of the ethical committee of Animal Research Center, National Cheng-Kung University. The ethical guidelines that were followed meet the standards required by the UKCCCR guidelines (Workman *et al*, 1998). To investigate lung metastasis, mice received 0.5–5×10^5^ of tumour cells in 0.1 ml PBS via the tail vein injection (i.v. injection). Metastatic lung tumours in mice were assessed under a dissecting microscopy. Organs were surgically obtained, fixed in 10% buffered formalin solution for paraffin block preparation or for flash-frozen in O.C.T. embedding medium (Miles Inc., Elkhart, IN, USA). Five-μM tissue sections were placed on poly-L-lysine-coated glass slides, fixed with 3.7% paraformaldehyde, and treated with 3% H_2_O_2_. Cells were stained with rat anti-NK mAb (DX5), rat anti-CD4 mAb (H129.19), rat anti-CD8 mAb (53-6.7) (PharMingen, San Diego, CA, USA), rat anti-granulocyte mAb (RB6-8C5), rabbit anti-Fas Ab (M-20) or rabbit anti-Fas ligand Ab (N-20) (Santa Cruz Biotechnology, CA, USA). Appropriate sheep anti-rat IgG or goat anti-rabbit IgG conjugated with peroxidase (Boehringer Manheim GmbH, Mannheim, Germany) was used as secondary antibodies. Peroxidase staining was developed by aminoethyl carbazole substrate kit (Zymed, San Francisco, CA, USA) and showed reddish-brown colour. Sections were counterstained with haematoxylin and mounted with glycerol gelatin.

### Detection of apoptotic cells in culture

Apoptotic cells become susceptible to merocyanine 540 (MC540, Sigma, St. Louis, MO, USA) binding due to alteration of surface membrane and can be detected by flow cytometric analysis (Reid *et al*, 1996; Yang *et al*, 1999). To stain apoptotic cells, cells of 3-day-old culture were harvested, suspended in PBS containing 0.1% BSA and stained with 1 mg ml^−1^ MC540 for 10 min in the dark. Subsequently, cells were washed once with PBS and subjected to flow cytometric analysis (FACScan, Becton Dickinson, Mountain View, CA, USA) with a gate set to examine a total of 10^4^ cells. Apoptotic cells in tumour nodules were detected by TUNEL labelling detecting free 3′-OH groups in fragmented DNA *in situ* (ApopTag-peroxidase *in situ* apoptosis detection kit, Oncor, MD, USA). Paraffin-embedded, slide-mounted tissue sections were deparaffinised and treated with proteinase K followed by 3% H_2_O_2_. After nick end labelling with digoxigenin-deoxyuridine triphosphate by terminal deoxynucleotidyl transferase, immunostaining was performed using peroxidase-conjugated anti-digoxigenin Ab. Apoptotic cells were visualised with diaminobenzidine substrate and became brown colour.

### Depletion of CD4^+^, CD8^+^ cells and granulocytes

Ascitic fluids were generated from hybridomas GK1.5, 2.43 and RB6-8C5 secreting rat monoclonal antibodies for antibodies against mouse CD4, CD8 and granulocyte marker (Ly-6G), respectively (Staats *et al*, 1991; Tumpey *et al*, 1996). The CD4- and CD8-specific Abs were further purified by affinity chromatography on a protein G-sepharose column (Pharmacia, LKB Biotechnology, Piscataway, NJ, USA) and adjusted to a final concentration of 3 mg ml^−1^. Protocols modified from Staats *et al* (1991) was used to deplete CD4^+^ or CD8^+^ cells, so mice were given 100 μg anti-CD4 or anti-CD8 Ab by intraperitoneal (i.p.) injection on day −2. Booster anti-CD4 or anti-CD8 Ab was given twice on days 7 and 14. Depletion of granulocytes was achieved by a serial of i.p. injections with anti-granulocyte Ab according to a modified protocol as previously reported (Tumpey *et al*, 1996) as follows: 100 μl on −5 h; 100 μl on day 3; 150 μl on day 5; 200 μl on days 7 and 9; 250 μl on days 12 and 15. Control mice received purified rat IgG (ICN, Pharmaceuticals Inc. Cappel, OH, USA) following similar protocol. Spleen cells were stained with anti-CD4 (H129.19, FITC-conjugated; PharMingen) or anti-CD8 (53-6.7, PE conjugated; PharMingen) on days 6 or 18 to determine the extent of T cell depletion. Reduced number of granulocytes in peripheral blood in granulocyte-depleted mice was also confirmed on days 6 or 18 by flow cytometric analysis using FITC-conjugated antibody (RB6-8C5; PharMingen).

### Statistic analysis

Results were analysed by Student's *t*-test. Differences with *P*<0.05 were judged as significant.

## RESULTS

### Fas-L^ribozyme^-carrying cells

**Figure 1:**
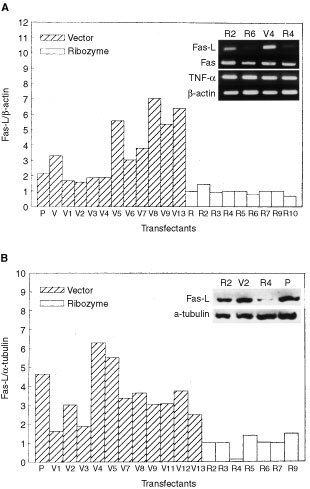
Decreased expression of Fas-L in Fas-L^ribozyme^-carrying cells. After transfection, cells were selected in geneticin-containing medium for 3 months. RT–PCR and Western blot analysis were performed as described in Materials and Methods. (**A**) The densities of ethidium bromide-stained RT–PCR products corresponding to Fas-L and β-actin were quantified by a densitometer. The ratio of Fas-L over β-actin represents the Fas-L expression in individual clones. The inserted photograph shows representative gels for RT–PCR-amplified products of Fas-L, Fas, TNF-α and β-actin. (**B**) The densities of protein bands corresponding to Fas-L and α-tubulin were quantified by a densitometer. The ratio of Fas-L over α-tubulin represents the amount of Fas-L level in individual clones. Representative Western blot presented in inserted photograph shows the expressions of Fas-L and α-tubulin. Vn: cells carrying pEGFP-N1 control plasmid; Rn: Fas-^ribozyme^-carrying cell clones; P: parental B16F10 cells.

**Figure 2:**
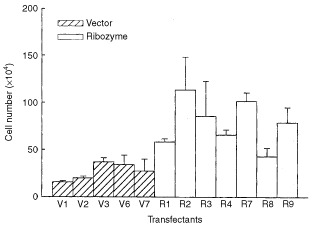
Growth rate of cells with or without Fas-L^ribozyme^. About 5×10^4^ cells were grown in regular 10% FCS/DMEM for 72 h. Cell number was then counted. Clones carrying EGFP-N1 plasmid (Vn); clones carrying Fas-L^ribozyme^ (Rn). Data represent means±s.d. of three independent experiments.

**Figure 3:**
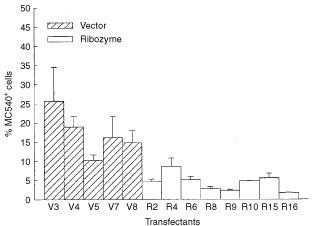
Apoptosis in 3-days culture of cells with or without Fas-L^ribozyme^. Cells, with (Rn) or without (Vn) Fas-L^ribozyme^, were grown in 10% FCS/DMEM for 72 h. Apoptotic cells were stained by MC540. Cells with FL2>230 were judged as MC540-positve and apoptotic. Data represent means±s.d. of three independent experiments.

After DNA transfection followed by antibiotic selection, bulk cultures and randomly chosen cell clones were established. Most of those stable clones emitted green fluorescence under UV light and showed similar morphology as parental B16F10 cells. Cells carrying pEGFP-N1 plasmid were named V for bulk culture and Vn for stable clones; cells carrying Fas-L^ribozyme^ plasmid were named R for bulk culture and Rn for stable clones. Rn, Fas-L^ribozyme^-carrying clones, showed reduced expression of Fas-L to various extents both at transcriptional and translational levels (Figure 1a,b

). Fas-L^ribozyme^ did not affect the expressions of Fas and TNF-α in melanoma cells. Along with a reduction in Fas-L protein, most of those Rn cells grew slightly faster *in vitro* than Vn, cells carrying pEGFP-N1 control plasmid, did (Figure 2

). We further measured the spontaneous apoptosis in 3-day cultures, which presumably had more Fas/Fas-L engagement due to cell–cell contact in relatively confluent growth. In comparison with the enhanced growth rate, less apoptosis was observed in Rn than Vn as detected by MC-540 staining (Figure 3

).

### Effect of Fas-L^ribozyme^ on lung metastasis

Bulk cultures and several established cell clones have been used to evaluate the contribution of tumour Fas-L to lung metastasis in C57BL/6 mice. Mice began to develop grossly observable tumour nodules in the lung around 14–18 days after inoculation with 1×10^5^ of parental B16F10 cells. Similarly, metastatic tumour nodules were observed in mice who received stable cells of Vn, bulk culture or clones, at day 14 post-inoculation (Table 1

**Table 1:**
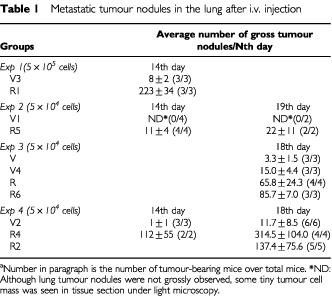
Metastatic tumour nodules in the lung after i.v. injection

). Rn produced more lung tumour nodules in mice than Vn did after day 14 post-inoculation (Figure 4

**Figure 4:**
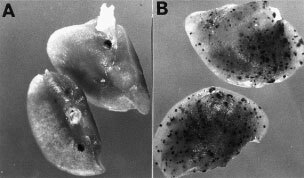
Tumour formation of cells with or without Fas-L^ribozyme^ in 8-week-old C57BL/6 mice. Approximate 5×10^4^ stable cells, vector control (V2; **A**) or Fas-L^ribozyme^ carrying cells (R4; **B**) were inoculated through tail vein. Lung was harvested to observe tumour nodules at 14 days post-inoculation.

). Tumour cells accumulated first in alveoli (day 7) and then expanded to alveolar sacs or near blood vessels (after 14 days).

### CD4^+^-, CD8^+^ cells- and granulocyte-depletion

**Figure 5:**
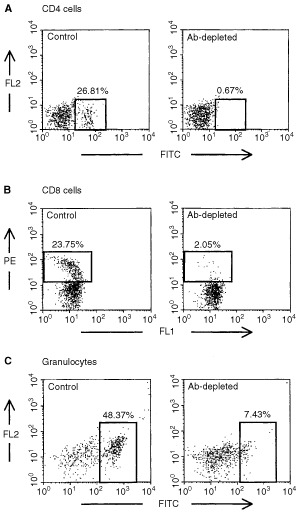
CD4^+^, CD8^+^ cells or granulocytes in antibody-treated mice. Spleenic CD4^+^- (**A**), CD8^+^ cells (**B**) or granulocytes (**C**) in circulating blood of mice were stained with antibodies for CD4, CD8 or granulocytes, respectively or control-Ig. Shown are representative dotplots.

**Figure 6:**
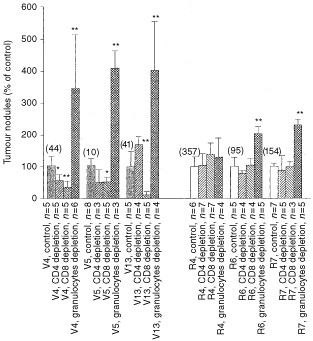
The formation of metastatic lung tumours in mice depleted for CD4^+^-, CD8^+^-cells or granulocytes. Vn: vector controls; Rn: Fas-L^ribozyme^-carrying cells. Values shown are average of three independent experiments. The numbers shown in parentheses over bars indicated the original total number of lung tumour nodule. *P<0.05; **P<0.01.

To explore the anti-tumour effect of immune cells *in vivo*, CD4^+^, CD8^+^ cells and granulocytes in mice were depleted by antibodies. The extents of depletions for CD4^+^ and CD8^+^ cells in the spleen were more than 90% (Figure 5A,B

). After depletion by RB6-8C5 antibody, the number of granulocytes in peripheral blood reduced to about 30% (Figure 5C). Figure 6

summarised the effects of CD4^+^-, CD8^+^ cells- or granulocyte-depletion on lung metastasis. Mice depleted of granulocytes showed an elevated susceptibility to lung metastasis (Figure 6). Particularly, the incidence of lung metastasis of controls in granulocytes-depleted mice at day 18 post-inoculation reached a degree similar to that of Rn in immune-competent mice. CD4^+^- or CD8^+^ cells-depletion did not affect, or even slightly suppress, lung metastasis of vector controls. Among the Fas-L^ribozyme^-carrying clones, R4 cells generated the highest number of lung tumour nodules in this study and its metastatic ability was not further increased in mice receiving antibodies against CD4^+^- CD8^+^ cells- or granulocytes.

### Immunohistochemical studies on the expression of Fas-L, tumour infiltrating cells and apoptosis in tumours

**Figure 7:**
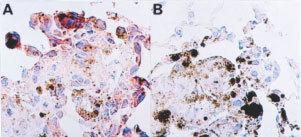
Representative immunohistochemical staining of Fas-L protein on tumour nodules. Lung tumour nodules of vector control (**A**) or Fas-L^ribozyme^-carrying cells (**B**) were surgically obtained at 18 days post-inoculation and fixed in 4% paraformaldehyde. Cryosection was immunostained with antibody specific for Fas-L. Fas-L-positive cells show a reddish-brown colour. Tumour cells of Vn were stained more intensively than those of Rn.

A reduced expression of Fas-L was immunohistochemically detected in metastatic lung tumours of Rn compared to that of Vn (Figure 7

). Histological examination revealed that small tumour cell mass were formed in the lung 96 h post-inoculation of tumour cells. Parental and control melanoma cells accumulated around terminal bronchioles, alveolar ducts or alveolar sacs. Rn accumulated around pulmonary veins and bronchioles. Similar to parental cells, the cell lines established in this study did not develop tumours in organs including liver, kidney, and spleen by i.v. inoculation at day 18 post-inoculation (data not shown).

Granulocytes accumulated in the lung very soon, within 48–96 h (Table 2

**Table 2:**
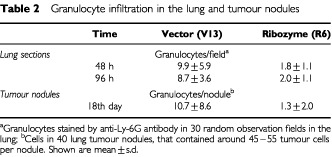
Granulocyte infiltration in the lung and tumour nodules

). Intensive granulocyte infiltration in the bronchioles, alveoli and alveoli sacs was observed in mice inoculated by Vn (Figure 8A

**Figure 8:**
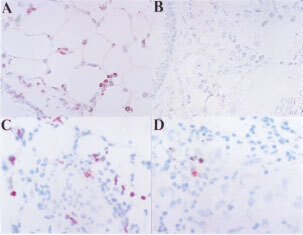
Tumour infiltrating granulocytes. Tumour formation and tissue preparation were performed as the procedures described in Figure 4. Cryosections were immunostained with RB6-8C5 antibody recognising granulocytes. Positive cells show a reddish-brown colour. (**A** and **C**) vector control; (**B** and **D**) Fas-L^ribozyme^-carrying cells. (**A** and **B**): lung samples of mice 96 h post-inoculation; (**C** and **D**) tumour nodules of 18 days post-inoculation.

). In tumour nodules formed after 14–18 days post-inoculation, cell infiltration was reduced. A small number of infiltrating cells including CD4^+^, CD8^+^ and NK cells were scattered near most tumour foci (data not shown). However, tumour foci with some infiltrated cells mostly comprised granulocytes were also seen (Figure 8C), although with low frequency. Fas-L^ribozyme^ reduced significantly the number of infiltrating granulocytes in tumour nodules of 96 h and 18 days after cell inoculation (Figure 8B,D), but not the numbers of CD4^+^, CD8^+^ and NK cells.

**Figure 9:**
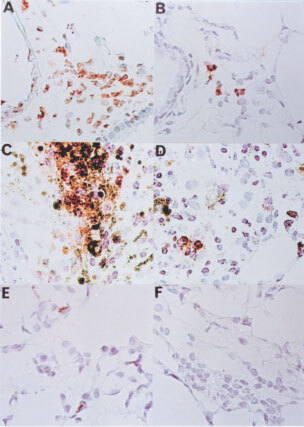
Apoptotic cells in tumour nodules. Apoptotic cells were detected by TUNEL-staining as described in Materials and Methods. Apoptotic cells revealed brown colour. (**A** and **B)** lung samples of mice 96 h post-inoculation, (**C** and **D**) tumour nodules of 18 days post-inoculation, (**E** and **F**) lung samples (96 h post-inoculation) of mice depleted for granulocytes; (**A**, **C** and **E**) vector control; (**B**, **D** and **F**): Fas-L^ribozyme^-carrying cells.

Massive apoptotic cells were observed in the lung of mice received Vn cells in 48–96 h (Figure 9A

) post-inoculation. Apoptosis still occurred frequently in tumour nodules formed by Vn after 14 days post-inoculation (Figure 9C). Furthermore, quite a few death cells in tumour nodules were morphologically tumour origin and TUNEL-staining positive. Apoptosis was ameliorated significantly in tumour nodules of Rn (Figure 9B,D). We further evaluated the apoptosis in granulocyte-depleted mice. Few cells were TUNEL-positive in the tumour nodules obtained from granulocyte-depleted mice inoculated with Rn or Vn for 96 h (Figure 9E,F).

## DISCUSSION

The application of Fas-L^ribozyme^ effectively and specifically suppressed the expression of Fas-L in B16F10 cells as revealed by RT–PCR, Western blot and immunohistochemistry. Transfectants carrying Fas-L^ribozyme^ grew slightly faster *in vitro* with better viability than vector controls. Since Fas-L^ribozyme^ did not alter the expressions of Fas and TNF-α, a suppression of the Fas-L by Fas-L^ribozyme^ resulting in less Fas/Fas-L ligation should account for the reduced apoptosis *in vitro*. The suicidal destruction of tumour cells has been reported in Fas signal-sensitive tumours after transfer of Fas-L gene (Arai *et al*, 1997; Walker *et al*, 1998). In addition, delivering Fas-L^ribozyme^ into human glioma or Ras-activated NIH3T3 malignant cell lines also reduced apoptosis in those cells (Chio *et al*, 2001, our unpublished data). It seems that the Fas/Fas-L suicidal effect could occur widely in different malignant cells. Although a growth-promoting reverse signalling for lymphocytes through Fas-L has been suspected (Suzuki and Fink, 1998), down-regulation of Fas-L in melanoma cells caused an increase in growth rate *in vitro* indicating that Fas-L did not elicit such reverse signalling in melanoma.

We then further evaluated the lung metastatic potential of Fas-L^ribozyme^-transfectants in C57/BL6 mice. Down regulation of Fas-L by Fas-L^ribozyme^ drastically enhanced lung metastasis that was correlated with reductions in both apoptotic tumour cells and granulocytic infiltration. The recruitment of granulocytes in the Fas-L-positive tumours can be attributed to the proinflammatory effect of Fas-L that has been observed in several studies using ectopic Fas-L expressing cells (Seino *et al*, 1997; Chen *et al*, 1998). Alternatively, apoptotic body generated due to suicidal destruction by itself has also been demonstrated to be a potent chemotactic agent for cell recruitment (Horino *et al*, 1998). When phagocytes engulf apoptotic body, they can effectively initiate T cell immunity (Chattergoon *et al*, 2000). In addition to granulocytes, a small number of CD4^+^, CD8^+^, or NK cells were found near/around the tumours, but the pattern of these cells was not affected by Fas-L^ribozyme^ indicating that infiltration of these immune cells involved other chemotatic signal.

It is noteworthy that the tumorogenic capacity of Vn cells did not positively correlate with their absolute Fas-L amount suggesting that a critical amount of Fas-L protein exists to fully manifest the metastasis-inhibiting effect of Fas-L. When the amount of Fas-L on tumour cells is above the threshold, the effect of Fas-L reaches maximal. Only when the tumour Fas-L is in a range below the threshold, a trend of dose-dependent inhibition on lung metastasis of B16F10 was observable. For example, R4, a clone expressing very low amounts of Fas-L protein, formed the highest number of tumour nodules among all clones tested and that could not be further enhanced by granulocyte depletion. Besides, other uncharacterised factors may also contribute to tumour metastasis, which would attenuate minor differences in tumorigenicity between clones having litter difference in Fas-L expression and diminish the expected dose effect. As mentioned earlier, loss function of Fas has been linked to metastatic progression (Owen-Schaub *et al*, 1998). Similarly, we observed in this study that lung metastasis was negatively correlated with the Fas/Fas-L suicidal destruction occurred in melanoma cells *in vitro* and the appearance of apoptotic cells in lung tumour nodules. However, several lines of evidence showed that the autocrine suicidal Fas/Fas-L interaction was not the major limiting factor, at best acted only as an initiator, for the Fas-L associated apoptosis *in vivo* and for the reduced potential in metastasis. First, in the absence of granulocytes, the expression of Fas-L on tumour did not inhibit tumour metastasis. High Fas-L expressing cells, though showing high apoptosis *in vitro*, could efficiently develop lung tumours in granulocytes-depleted mice to a degree as Fas-L^ribozyme^-carrying cells did. Second, the apoptosis in tumour nodules obtained from granulocyte-depleted mice was drastically reduced, evidence of a granulocyte-dependant apoptosis in tumour. In these experiments, the suicidal Fas/Fas-L signal in cells was not altered. Therefore, tumour cell death is indeed a restriction factor for lung metastasis. However, the death signal is not directly due to the suicidal Fas/Fas-L signal. In addition, neutrophils isolated from peritoneal do kill tumour cells *in vitro*, though with different efficiency depending on whether they carrying Fas-L^ribozyme^ or not (our unpublished data). Thus, the recruited granulocytes mediate primarily the destruction of metastatic tumour cells in the lung.

A pivotal role of cells of innate immunity in tumour combat has previously been recognised in several tumours. The action of tumour-suppressive Th1 cells through CpG DNA is granulocyte-dependent (Egeter *et al*, 2000). Neutrophils, but not T cells, mediate the primary rejection of Fas-L-overexpressing, Fas-negative tumour cells *in vivo* (Seino *et al*, 1997). Our study provides firm evidence for the anti-tumour effect of granulocytes in lung metastasis. During the lung metastasis, T cells have few impacts on tumour control. Depletions of CD4^+^ or CD8^+^ cells did not affect (clone V3) or even slightly inhibit (clone V4) the progression of tumours having high Fas-L. Recent studies have also demonstrated that T cells specific for tumour antigens can become actively tolerated during progression of tumours (Mackensen *et al*, 1993; Adler *et al*, 1998). It is possible that altered T cells may produce a distinct profile of cytokine productions, which in turn stimulate tumour formation (Medvedev *et al*, 1997; Mori *et al*, 1997).
